# GABAergic Alterations in Neocortex of Patients with Pharmacoresistant Temporal Lobe Epilepsy Can Explain the Comorbidity of Anxiety and Depression: The Potential Impact of Clinical Factors

**DOI:** 10.3389/fncel.2014.00442

**Published:** 2015-01-05

**Authors:** Luisa Rocha, Mario Alonso-Vanegas, Iris E. Martínez-Juárez, Sandra Orozco-Suárez, David Escalante-Santiago, Iris Angélica Feria-Romero, Cecilia Zavala-Tecuapetla, José Miguel Cisneros-Franco, Ricardo Masao Buentello-García, Jesús Cienfuegos

**Affiliations:** ^1^Department of Pharmacobiology, Center for Research and Advanced Studies of the National Polytechnic Institute (CINVESTAV), Mexico City, Mexico; ^2^National Institute of Neurology and Neurosurgery “Manuel Velasco Suarez”, Mexico City, Mexico; ^3^Unit for Medical Research in Neurological Diseases, National Medical Center, Mexico City, Mexico

**Keywords:** GABA receptors, G-protein, temporal lobe epilepsy, temporal neocortex, anxiety, depression

## Abstract

Temporal lobe epilepsy (TLE) is a chronic neurodegenerative disease with a high prevalence of psychiatric disorders. Temporal neocortex contributes to either seizure propagation or generation in TLE, a situation that has been associated with alterations of the γ-amino-butyric acid (GABA) system. On the other hand, an impaired neurotransmission mediated by GABA in temporal neocortex has also been involved with the pathophysiology of psychiatric disorders. In spite of these situations, the role of the necortical GABA system in the comorbidity of TLE and mood disorders has not been investigated. The present study was designed to identify alterations in the GABA system such as binding to GABA_A_ and GABA_B_ receptors and benzodiazepine site, the tissue content of GABA and the expression of the mRNA encoding the α1–6, β1–3, and γ GABA_A_ subunits, in the temporal neocortex of surgically treated patients with TLE with and without anxiety, and/or depression. Neocortex of patients with TLE and comorbid anxiety and/or depression showed increased expression of the mRNA encoding the γ2-subunit, reduced GABA_B_-induced G-protein activation in spite of elevated GABA_B_ binding, and lower tissue content of GABA when compared to autopsy controls. Some of these changes significantly correlated with seizure frequency and duration of epilepsy. The results obtained suggest a dysfunction of the GABAergic neurotransmission in temporal neocortex of patients with TLE and comorbid anxiety and/or depression that could be also influenced by clinical factors such as seizure frequency and duration of illness.

## Introduction

It is known that a high percentage of patients with epilepsy have comorbid interictal psychiatric disorders (Briellmann et al., 2007; Kanner, 2011). This situation can be explained by pathophysiological mechanisms associated with specific neurotransmitters in the brain areas involved in both, epilepsy and psychiatric conditions (Rocha et al., 2014).

Temporal neocortex is a paralimbic structure that belongs to a visceromotor system associated with mood, emotions, and visceral reactions to emotional stimuli (Beauregard et al., 1988; Partiot et al., 1995; Ongür et al., 1998; Drevets et al., 2008). Neuroanatomical studies in non-human primates indicate that temporal cortex is involved in the sensory integration as well as codification of affective characteristics of stimuli (Ongür and Price, 2000; Saleem et al., 2007). Abnormalities in gray matter volume and glucose metabolism have been detected in the temporal neocortex of patients with mood disorders (Ongür et al., 2003). Temporal neocortex of patients with depression presents overactivation (Sheline et al., 2009) as well as abnormalities in cell communication and signal transduction systems identified by transcriptional profiling (Aston et al., 2005). Alterations of γ-amino-butyric acid (GABA) system in temporal neocortex have been proposed to participate in the pathophysiology of mood disorders (Nikolaus et al., 2010).

On the other hand, abnormal neurotransmission mediated by GABA system in the cortex has been suggested to play an important role in seizure generation and/or propagation (Chagnac-Amitai and Connors, 1989) as well as in the neuronal overactivation detected in this brain of patients with temporal lobe epilepsy (TLE) (Avoli et al., 1995; Koepp et al., 2000; Teichgräber et al., 2009). Nevertheless, studies aimed to identify alterations on GABA receptor binding in the temporal neocortex of patients with pharmacoresistant TLE have shown dissimilar results (la Fougère et al., 2009).

Although previous studies suggest that GABA disturbances in the temporal neocortex participate in the pathophysiology of TLE and comorbid mood disorders (Kondziella et al., 2007), this idea has not been investigated. The present study was focused to evaluate a possible association between alterations in the GABAergic system in the temporal neocortex of patients with pharmacoresistant TLE and comorbid anxiety and/or depression. Experiments were designed to analyze the binding to GABA_A_ receptors involved in tonic ([^3^H]-Muscimol) and phasic ([^3^H]-Flunitrazepam) neurotransmission, as well as GABA_B_ receptor binding. We also evaluated the G-protein activation mediated by GABA_B_ receptors, the tissue content of GABA and the mRNA expression of some GABA_A_ receptor subunits. Values obtained were correlated with clinical data to identify those clinical factors that could be involved in specific alterations of the GABAergic system.

## Materials and Methods

### Patients criteria and surgical samples

Biopsy samples of temporal neocortex were obtained from 26 patients with diagnosis of pharmacoresistant TLE: 16 patients with mesial TLE, nine patients with TLE secondary to tumor or lesion, and one patient with dual pathology namely mesial TLE and tumor. All patients underwent epilepsy surgery after been submitted to an extensive pre-surgical evaluation according to the protocol of the Epilepsy Surgery Program of the National Institute of Neurology and Neurosurgery “Manuel Velasco Suarez,” in Mexico (Table S1 in Supplementary Material).

The pre-surgical evaluation consisted of neurological evaluation, electroencephalogram (EEG) and video-EEG recordings, single photon emission computed tomography (SPECT) or positron emission tomography (PET), neuropsychological and neuropsychiatric evaluation, and magnetic resonance imaging (MRI). MRI findings were in concordance with those found in EEG recordings.

During the neurological evaluation, prevalence of depression and anxiety disorders was established using the Structured Clinical Interview for DSM-IV Axis I (SCID-I) (First et al., 1999) applied by a Psychiatrist, who was blinded to the epilepsy diagnosis. Spanish version of the Hospital Anxiety and Depression Scale (HADS) were applied to all patients to identify symptoms of anxiety and/or depression. HADS has been previously validated in a Spanish population (Herrero et al., 2003; Gómez-Arias et al., 2012). HADS scale considers symptoms over the previous week and is not affected by coexisting general medical conditions. Patients with other psychiatric or somatic disturbances interfering with mood disorders, such as addiction, were excluded from the present study.

The neurosurgeon (Mario Alonso-Vanegas) carried out all the surgeries. Patients with mesial TLE underwent epilepsy surgery using a T2 or T3 transtemporal approach and guided by electrocorticographic (ECoG) signals recorded from the brain surface (4 × 8-electrode grids, Ad-Tech, Racine, WI). The epilepsy surgery consisted of unilateral amygdalo-hippocampectomy that included removal of the uncus and parahippocampal gyrus, and the tailored resection of T2 and T3 (San-Juan et al., 2011). Patients with tumor or lesion, with and without TLE had a similar surgical procedure unaided by ECoG recordings, with standard temporal neocortical resection. In these patients, amygdalo-hippocampectomy was performed depending on the localization of the lesion or tumor and neuropsychological findings. After resection, T2 and T3 gyri were immediately frozen in milled dry ice and kept at −70°C until processing. When the tumor or lesion was restricted to the temporal neocortex, samples from the margins of the lesion were used for present study. The protocol did not include biopsies with tumor, cortical malformations or any cortical alteration identified by neuropathological evaluation.

The present study was approved by the scientific committees of the institutions involved in this research and informed authorization and consent were obtained from each patient.

### Autoradiography experiments

Previous studies specify that receptor binding and guanosine 5′-O-[γ-thio [^35^S]] triphosphate ([^35^S]GTPγS) binding stimulation by selective agonists to GABA_B_ receptors are preserved for several hours after death (González-Maeso et al., 2000), while longer post-mortem delay has been associated with increased binding to benzodiazepine (BDZ) sites (Whitehouse et al., 1984). Considering this information, binding values acquired from autopsies of six men who died as consequence of diverse causes without clinical data of neurologic or psychiatric disorders with a post-mortem interval of 2–14 h were compared to those obtained from the patients with pharmacoresistant TLE (Table S1 in Supplementary Material). T2 and T3 gyri were dissected at the time of the autopsy and quickly kept at −70°C. For each autoradiography assay, tissue samples of the different patients and autopsies were processed together in order to reduce the experimental variability.

#### Preparation of tissue sections

Frozen sections of 20 μm were cut in a cryostat, thaw-mounted on gelatin-coated slides, and kept again at −70°C. Serial and parallel sections were obtained from each biopsy/autopsy for subsequent quantitative and functional autoradiography procedures.

#### Quantitative autoradiography

Table 1 includes a summary of the different protocols for the quantitative autoradiography experiments. Brain sections were removed from the freezer, dried in a stream of cool air, and immediately washed to eliminate endogenous ligands. Then, sections were incubated in a solution with the specific ligand labeled with tritium ([*^3^*H]), in presence or absence of a non-labeled specific ligand. The specific binding values were established from the difference of values obtained from both experimental conditions. Incubation was concluded with two consecutive washes in buffer solution and a final rinsed with distilled water was carried out for 2 s at 4°C. Slices were quickly dried in a mild steam of cold air.

**Table 1:** **Conditions for quantitative autoradiography experiments**.

Binding	Ligand (nM) and SA	Buffer pH 7.4	Incubation	Exposition (RT)	Non-labeled ligand
GABA_A_	[^3^H]-Muscimol (20 nM) 20 Ci/mmol	Tris Citrate (50 mM)	45 min at 4°C	8 weeks	GABA (10 μM)
GABA_B_	[^3^H]-CGP54626 (4 nM) 30 Ci/mmol	Tris–HCl (50 mM) and CaCl_2_ (10 mM)	90 min at 22°C	12 weeks	CGP55845 (100 μM)
Benzodiazepines	[^3^H]-Flunitrazepam (2 nM) 85.2 Ci/mmol	Tris–HCl (170 mM)	45 min at 4°C	3 weeks	Clonazepam (1 μM)

*SA, specific activity; RT, room temperature*.

Slices from patients and autopsies as well as [*^3^*H] standards (Amersham) were arranged together in X-ray cassettes and all of them were exposed to [*^3^*H]-sensitive film (Kodak MR) at 22°C. After the appropriate exposure time (see Table 1), the film was developed at 18–20°C in Kodak D19 developer and fast fixer. Optical densities of cortical layers of each tissue sample were evaluated in three different sections using the JAVA Jandel image analysis software. Temporal neocortex was subdivided for autoradiographic analysis into an outer layer (cortical layers I and II), middle layer (cortical layers III and IV), and an inner layer (cortical layers V and VI). The distribution of receptor binding sites, as revealed by optical densities of the autoradiograms obtained by outlining each layer, was matched to the cortical layers visualized on the sections counter-stained with 0.5% Cresyl Violet (Figure 2). Finally, the generation of a standard curve using the optical density values of the standards, the specific activity of each [*^3^*H]-labeled ligand, and tissue thickness (20 μm) was used to convert radioactivity values in fmol/mg of protein.

#### Functional autoradiography

Sections were washed in Tris buffer (50 mM Tris–HCl, 3 mM MgCl_2_, 0.2 mM EGTA, 100 mM NaCl, pH 7.4 at 25°C for 10 min), then incubated in the assay buffer containing 2 mM GDP (25°C for 15 min). GABA_B_-induced G-protein activation was evaluated in sections subsequently incubated in the same assay buffer with 2 mM GDP and 0.04 nM [^35^S]GTPγS (25°C for 2 h) in the presence of baclofen (100 μM), a GABA_B_ receptor agonist. In parallel sections, the effects of baclofen were evaluated in the presence of a GABA_B_ antagonist (CGP55845A, 10 mM). Basal binding was determined in sections incubated in similar conditions, but lacking agonist and antagonist drugs. Thereafter, slices were washed twice for 2 min each in assay buffer (4°C, pH 7.4) and once in distilled water (4°C). Sections from autopsies and patients were dried overnight and exposed to film (Kodak-MR) for 5 days at 22°C in X-ray cassettes containing [^14^C] microscales (American Radiolabeled Chemicals, Inc.). Optical density analysis of the different cortical layers was carried out as previously described for quantitative autoradiographic experiments. Results obtained from the different assays were expressed as nanocuries of [^35^S] per milligram of tissue. Net agonist-stimulated [^35^S]GTPγS binding was calculated in percentage by subtracting basal binding from agonist-stimulated binding.

### Semiquantitative RT-PCR Analysis

Expression levels of human GABA_A_ receptor subunit mRNAs (α1- to α6-, β1- to β3-, and γ1- to γ3-subunits) were determined from resected human tissues of autopsies and patients with pharmacoresisant TLE by semiquantitative RT-polymerase chain reaction (PCR) procedure. For this purpose, a fragment was obtained from each brain sample of patients with epilepsy and from the autopsies. The fragmentation was done maintaining the tissue frozen, a situation that allowed to preserve the mRNA.

Total RNA was isolated using the TriPure isolation Reagent (ROCHE, USA) according to the manufacturer’s instructions. Reverse transcriptase (RT) of 3 μg of total RNA was synthesized to single-stranded cDNA using random primers (Promega, USA) and 200 U of M-MuLV RT (New England Biolabs, USA) in a total reaction volume of 10 μl. The RT reaction was performed for 10 min at 25°C, 50 min at 37°C and 15 min at 70°C according to the manufacturer’s instructions. After RT, 20 μl of ultrapure-grade water was added to the final reaction. Gene amplifications were performed using 1 μl of diluted cDNA, 0.2–0.4 μl of 60 mM MgCl_2_, 0.2 μl of dNTP mix solution (Promega, USA), 0.2 μl of each primer pair (10 mM) and 0.2 μl of Taq DNA polymerase (Invitrogen, USA) in a total volume of 10 μl. The final MgCl_2_ concentration depended on each gene evaluated. The PCR was carried out in a DNA Thermal Cycler (Bio-Rad, USA) with a cycle program of 94°C for 3 min, 30–35 cycles at 94°C for 30 s, 54–58°C for 20–30 s, 72°C for 30–40 s, and one cycle of 72°C for 10 min. The cycle program was performed until the exponential phase was achieved; other PCR conditions were performed individually. The PCR products were separated by electrophoresis in a 2% agarose gel (Invitrogen) in TAE buffer at 75 V. The gels were captured and evaluated in an Alpha Innotech corporation IS-1000 digital imaging system, using EtBr (0.6 g/ml by gel) under UV light. Genomic DNA contamination was checked by carrying samples through a PCR procedure without adding reverse transcriptase.

The bands from images were analyzed using the NIH Image J system version 1.46 (http://imagej.nih.gov/ij/) and quantified as values of integrated density. The relative value of each gene was a ratio between the expression of each gene and β-actin as control.

### Tissue content of GABA

Evidence exists indicating a significant degradation by proteolysis of the GABA-synthesizing enzyme (GAD) and reduced GABA tissue content in the brain tissue within hours after death (Lowe et al., 1988; Martin et al., 2003). Since this condition can represent a potential problem in the evaluation of the tissue content of GABA in autopsy samples, temporal neocortex of six patients (four men and two women) submitted to surgery with diagnosis of cerebral tumor without epilepsy was used as control tissue to be compared with values obtained from patients with pharmacoresistant TLE (Table S1 in Supplementary Material).

Gray matter (50–100 mg) of each brain sample was thawed and manually homogenized in perchloric acid (0.1 M, J. T. Baker). The homogenates were centrifugated at 13,200 rpm (15 min at 4°C) using a centrifuge (Eppendorf 5415R). Samples of the supernatant (100 μl) previously filtered (Nalgene filters of 0.45 μm) were suspended in 0.1 M perchloric acid in a 1:250 proportion. Subsequently, 20 μl of the filtered supernatant were mixed with 6 μl of o-phthalaldehyde (OPA) and agitated for 30 s. Two minutes later, the mixture was injected into the solvent stream of a high performance liquid chromatography (HPLC) system. For GABA quantification, the procedure required that OPA-amino acid were separated on a reversed-phase 3.9 × 150 mm column (Nova-Pack, 4 μm, C18, Waters^®^) with solution A (sodium acetate dissolved in 90% miliQ water and 10% methanol; pH 5.75 with glacial acetic acid) as aqueous solvent and solution B as the other mobile phase (20% solution A and 80% methanol; pH 6.75 with glacial acetic acid) at a flow rate of 0.5 ml/min. Content of GABA was determined with fluorescent detection (Waters^®^ model 474) by peak height measurements against standard solutions (Kendrick et al., 1988).

The pellets obtained from the centrifugation process were used to determine the amount of proteins (Lowry et al., 1951), a situation that allowed expressing in micromoles per milligram of proteins the values resulting from the fluorometric HPLC procedure.

### Statistical analysis

The results obtained were expressed as mean ± SE and analyzed employing ANOVA test and Bonferroni *post hoc* test. Pearson’s correlation coefficients were estimated to establish the potential impact of clinical factors such as patient’s age, age at seizure onset, duration of epilepsy, and seizure frequency on the GABAergic system.

## Results

### Clinical characteristics

Anxiety and depression were detected in 10 patients with TLE. Their clinical data were as follows (mean ± SE): age of patients, 36.4 ± 3 years (ranged from 24 to 48 years); age at seizure onset, 14.2 ± 4.5 years; years of epilepsy duration, 22.2 ± 3.3; and seizures per month, 16.4 ± 4.5. Regarding pharmacological therapy that these patients received during the epilepsy process and pre-surgical period, the following information was identified: (a) 90% (*n* = 9) received polytherapy [from 4 to 10 antiepileptic drugs (AEDs)]; (b) 80% (*n* = 8) were treated with AEDs that could induce psychiatric adverse effects, such as depression (levetiracetam, primidone, zonisamide, topiramate, and phenobarbital); (c) 90% (*n* = 9) received 2 or more AEDs with GABAergic properties (clobazam, clonazepam, diazepam, levetiracetam, lamotrigine, valproic acid, phenobarbital, and primidone); and (d) 80% (*n* = 8) were treated with AEDs that could induce positive effects on the mood (valproic acid, carbamazepine, and gabapentin). Four patients (40%) had been previously diagnosed with depression and treated with antidepressant drugs (amitriptyline, duloxetine, sertraline, or fluoxetine) during 9 months to 6 years before the epilepsy surgery. Six patients (60%) were diagnosed with anxiety and/or depression during the pre-surgical evaluation, but they did not receive pharmacotherapy for these disorders (Table S1 in Supplementary Material).

Preclinical evaluation did not reveal neuropsychiatric comorbidity in 16 patients with pharmacoresistant TLE. Their clinical data (age, 30.6 ± 2.2 years old, ranged from 17 to 60 years old; age at seizure onset, 12.6 ± 2.1 years; years of epilepsy duration, 18.9 ± 3.1; and seizures per month, 16.5 ± 5.7) were not significantly different when compared to those patients with TLE and comorbid anxiety and/or depression. The pharmacological therapy that these patients received during the epilepsy process and pre-surgical period was similar to that administered to the patients with TLE and comorbid anxiety and depression: (a) 75% (*n* = 12) received polytherapy (from 3 to 7 AEDs); (b) 50% (*n* = 8) were treated with AEDs that may produce mood disorders; (c) 81% (*n* = 13) received 1 or more AEDs with GABAergic effects, and (d) 81% (*n* = 13) were treated with AEDs that induce a positive effect on the mood (Table S1 in Supplementary Material).

The mean age of patients with TLE with and without anxiety and/or depression was similar to the mean age of autopsies (39.5 ± 3.4 years, ranging from 29 to 51 years, *p* < 0.51) and patients with cerebral tumor without epilepsy and psychiatric disorders (39.6 ± 6.6 years, ranging from 25 to 63 years, *p* < 0.6) (Table S1 in Supplementary Material).

Nissl staining revealed a normal cytoarchitecture with no evident neuronal cell loss, cortical dysplasias, malformations, or tumor in the different tissues evaluated.

### Control and autopsy samples

**Figure 1:**
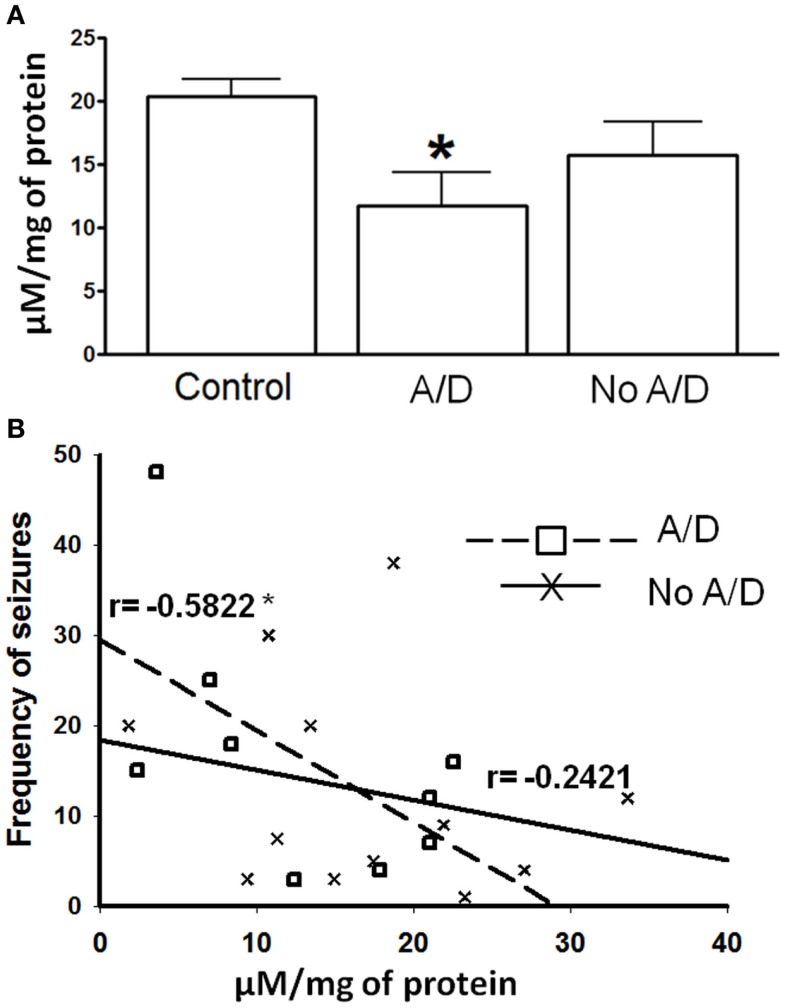
**(A)** γ-Aminobutyric acid (GABA) tissue content in temporal neocortex of control subjects and from patients with TLE with (A/D) and without anxiety and depression (No A/D). **(B)** Correlation between the frequency of seizures and the GABA tissue content in the temporal neocortex of patients with TLE with (
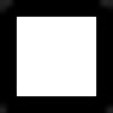
) and without (X) anxiety and depression. *r* indicates the Pearson coefficient value. Values are expressed as mean ± SE of micromoles per milligram of protein. *p < 0.05 vs. control.

**Figure 2:**
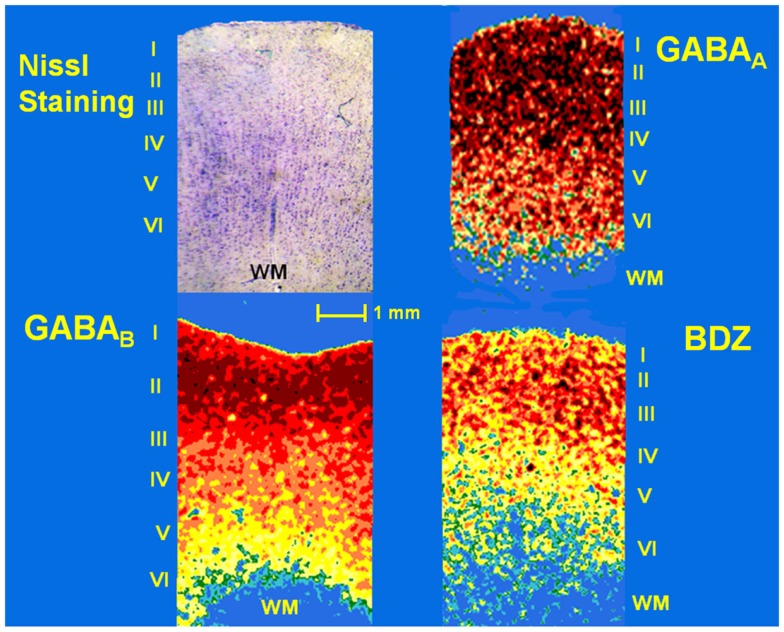
**Photomicrograph of a Nissl-stained section (showing layers I–VI) and pseudo-color autoradiographs obtained from an autopsy sample**. Autoradiographs show the distribution of binding to GABA_A_ and GABA_B_ receptors as well as benzodiazepine (BDZ) sites, labeled with [^3^H]-Muscimol, [^3^H]-CGP54626, and [^3^H]-Flunitrazepam, respectively. High binding appears as red and orange areas, low binding is indicated as yellow and green areas, whereas blue areas represent absence of binding.

Samples obtained from control patients with tumor without epilepsy presented 20.3 ± 1.3 μM/mg of protein of GABA tissue levels (Figure 1). In autopsy samples, binding to [^3^H]-Muscimol (GABA_A_ receptors) was widely distributed through the various cortical layers. Binding to [^3^H]-CGP54626 (GABA_B_ receptors) demonstrated a gradient across the cortical layers, showing the highest in outer layer. Binding to [^3^H]-Flunitrazepam (BDZ sites) was elevated in outer and middle layers (Figure 2 and Table 2). Functional autoradiography revealed [^35^S]GTPγS incorporation as consequence of the GABA_B_-induced G-protein activation (153% in layers I–II, 130% in layers III–IV, and 137% in layers V–VI) (Table 2).

**Table 2:** **[^3^H]Ligand binding to GABA_A_ and GABA_B_ receptors as well as BDZ site, and GABA_B_-induced G-protein activation in specific cortical layers of autopsies and samples of patients with temporal lobe epilepsy with (A/D) and without (No A/D) anxiety and depression**.

Binding	Group	Layers I–II	Layers III–IV	Layers V–VI
GABA_A_ receptor	Autopsy	468 ± 18	486 ± 29	359 ± 17
	A/D	465 ± 50	434 ± 40	365 ± 29
	No A/D	447 ± 40	463 ± 43	353 ± 32
GABA_B_ receptor	Autopsy	7747 ± 119	461 ± 61	262 ± 15
	A/D	927 ± 139	934 ± 134**	500 ± 88**
	No A/D	1051 ± 106	805 ± 58**	503 ± 42**
BDZ site	Autopsy	563 ± 91	648 ± 108	369 ± 62
	A/D	701 ± 136	784 ± 126	494 ± 80
	No A/D	1052 ± 115∗	1024 ± 78*	747 ± 56***
GABA_B_-induced G-protein activation	Autopsy	153 ± 28	130 ± 15	137 ± 10
	A/D	81 ± 14*	57 ± 12**	74 ± 9**
	No A/D	152 ± 24	110 ± 14	92 ± 9*

*Binding values are expressed as mean ± SME of fmol/mg of protein. The results of the GABA_B_-induced G-protein activation are expressed as mean ± SME of percentage of specific [^35^S]GTPγS binding with respect to basal value (100%). Statistical comparison was made using ANOVA and a *post hoc* Bonferroni test*.

* *p* < 0.05; **p < 0.01; ***p < 0.001, when compared with autopsy group.

In autopsy samples, the mRNA expression of the GABA_A_ receptor subunits was unrelated to the age of the subjects and time required to obtain the tissue. High mRNA levels were observed for subunits β1, β2, β3, and γ1, whereas subunits α1–6, γ2, and γ3 were less prominent.

### Patients with TLE

Patients with TLE without psychiatric disorders presented a non-significant decrease of the GABA tissue content (22%, *p* > 0.05) when compared to the autopsies. In contrast, the temporal neocortex of subjects with TLE and comorbid anxiety and/or depression showed lower tissue content of this amino acid (42%, *p* < 0.05), a situation that correlated with a higher seizure frequency (*r* = −0.5822, *p* < 0.05, Figure 1).

**Figure 3:**
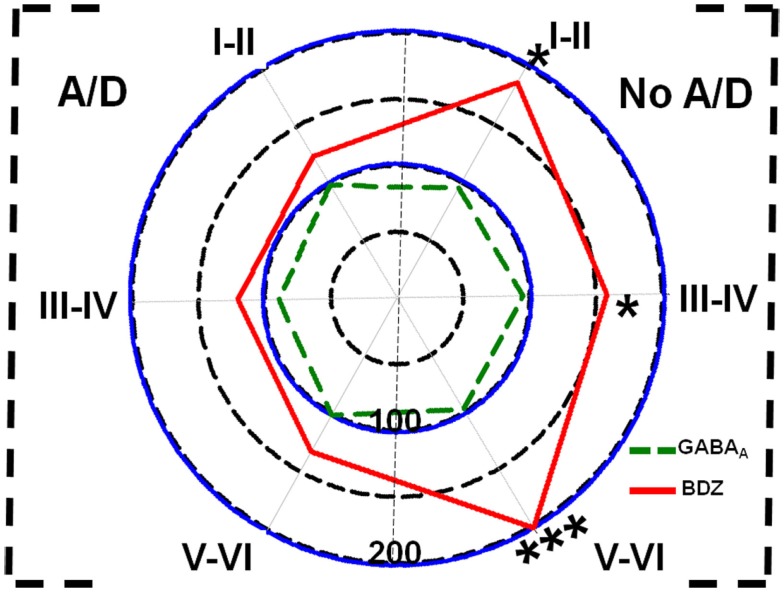
**Polar plots showing in percentage mean relative changes in binding to GABA_A_ receptors (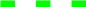 line) and benzodiazepine (BDZ) sites (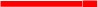 line) in temporal neocortex (layers I–II, III–IV, and V–VI) of patients with TLE with (A/D, left side) and without (No A/D, right side) anxiety and depression, with respect to autopsies (100%)**. *p < 0.05, ***p < 0.001.

**Figure 4:**
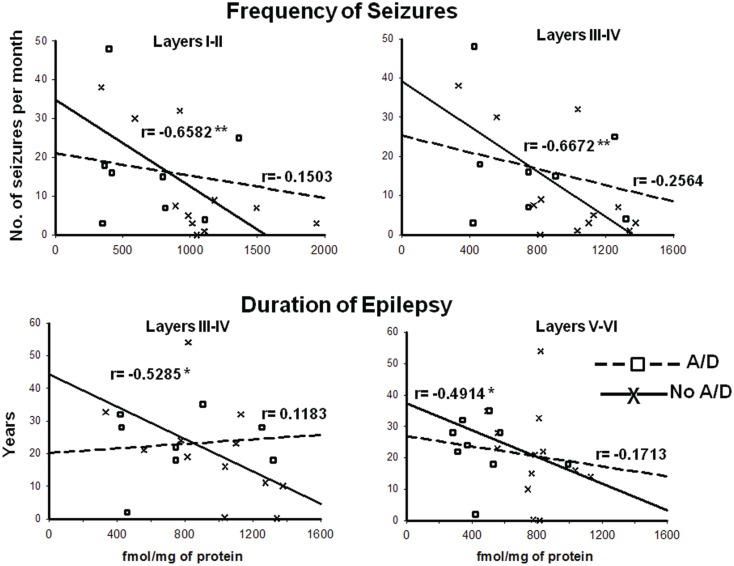
**Correlation between the frequency of seizures and benzodiazepine (BDZ) binding (cortical layers I–II and III–IV, upper panels), and duration of epilepsy and BDZ binding (cortical layers I–II and III–IV, lower panels) of patients with TLE with (
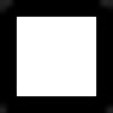
) and without (X) anxiety and depression**. *r* indicates the Pearson coefficient value. *p < 0.05, **p < 0.01.

Concerning binding evaluation, neocortex of patients with TLE with and without anxiety and/or depression did not show significant abnormalities in [^3^H]-Muscimol binding (Figure 3, Table 2). Patients without comorbid psychiatric disturbances showed higher [^3^H]-Flunitrazepam binding in all neocortex (layers I–II, 86%, *p* < 0.05; layers III–IV, 58%, *p* < 0.05; and layers V–VI, 103%, *p* < 0.001) compared to autopsies. In these patients, the higher [^3^H]-Flunitrazepam binding correlated with a lower seizure frequency (layers I–II, *r* = 0.6582, *p* < 0.01; and layers III–IV, *r* = 0.6672, *p* < 0.01) and a shorter duration of epilepsy (layers III–IV, *r* = 0.5285, *p* < 0.05; layers V–VI, *r* = 0.4914, *p* < 0.05) (Figure 4). [^3^H]-Flunitrazepam binding in neocortex of patients with TLE and anxiety and/or depression was not significantly different from autopsy group (Figure 3; Table 2).

RT-PCR experiments revealed high α4-subunit expression (114%, *p* < 0.05) in temporal neocortex of patients with TLE without mood disorders when compared with autopsies. The expression of the β-subunitis was not modified, whereas the γ2-subunit expression was increased in patients with (126%, *p* < 0.05) and without mood disorders (130%, *p* < 0.05).

**Figure 5:**
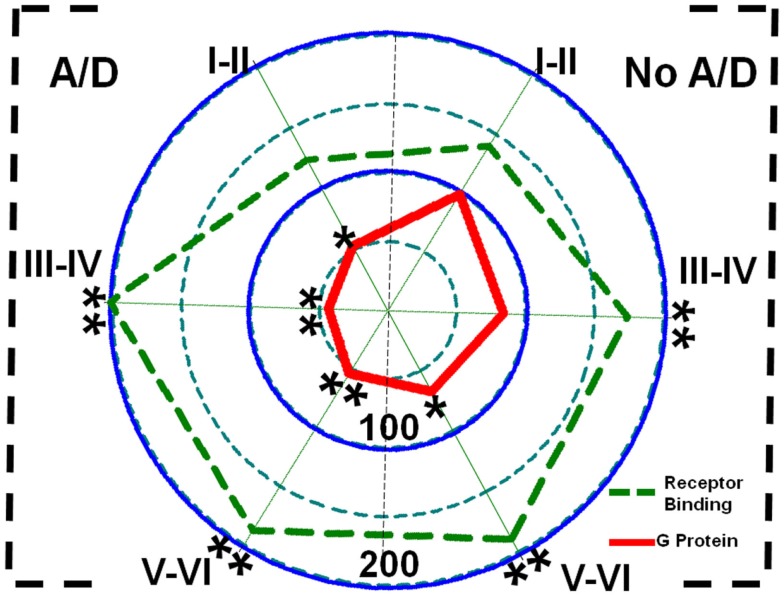
**Polar plots showing in percentage mean relative changes in GABA_B_ receptor binding (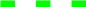 line) and GABA_B_-induced G-protein activation (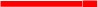 line) in the temporal neocortex (layers I–II, III–IV, and V–VI) of patients with TLE with (A/D, left side) and without (No A/D, right side) anxiety and depression, with respect to autopsies (100%)**. *p < 0.05, **p < 0.01.

[^3^H]-CGP54626 binding was increased in both, patients with (layers III–IV, 102%, *p* < 0.01; layers V–VI, 88%, *p* < 0.01) and without anxiety and/or depression (layers III–IV, 74%, *p* < 0.01; layers V–VI, 90%, *p* < 0.01) (Figure 5; Table 2). In contrast, [^35^S]GTPγS incorporation as consequence of activation of GABA_B_ receptors was lower in all cortical layers of patients with TLE and comorbid anxiety and/or depression (layers I–II, 47%, *p* < 0.05; layers III–IV, 56%, *p* < 0.01; and layers V–VI, 46%, *p* < 0.01) (Figure 5 and Table 2). In these patients, the lower GABA_B_-induced [^35^S]GTPγS incorporation correlated with a higher seizure frequency (layers III–IV, *r* = 0.7380, *p* < 0.05; layers V–VI, *r* = 0.8859, *p* < 0.01). Patients without psychiatric disturbances showed a lower GABA_B_-induced [^35^S]GTPγS incorporation restricted to deep layers (V–VI, 33%, *p* < 0.05) (Figure 5; Table 2), a situation that correlated with a higher seizure frequency (*r* = 0.5317, *p* < 0.05) and a longer duration of epilepsy (*r* = 0.4975, *p* < 0.05).

## Discussion

Previous reports indicate important changes of the GABAergic neurotransmission in the neocortex of patients with TLE (Avoli et al., 1995; Koepp et al., 2000; Teichgräber et al., 2009). However, data concerning abnormalities on the GABA receptor binding in the lateral temporal neocortex of patients with pharmacoresistant TLE are still controversial (la Fougère et al., 2009). The results obtained in the present study could explain this disagreement suggesting that specific alterations in the neurotransmission mediated by GABA in the temporal neocortex of subjects with pharmacoresistant TLE are associated with the comorbidity of anxiety and/or depression. Our results also indicate that clinical factors such as seizure frequency and epilepsy duration may play an important role in the disturbances of the GABAergic system in the temporal neocortex of patients with pharmacoresistant TLE and comorbid anxiety and/or depression.

[^3^H]-Muscimol binds to the same site on the GABA_A_ receptor complex as GABA itself (Frølund et al., 2002), and it labels extrasynaptic GABA_A_ receptors containing δ- and α4-subunits (Chandra et al., 2010). Extrasynaptic receptors are relevant sensors for ambient GABA and modulate the tonic inhibition (Nyitrai et al., 2006). We found no significant changes in [^3^H]-Muscimol binding in the neocortex of patients with TLE, regardless of the presence of anxiety and/or depression, suggesting that the binding to extrasynaptic GABA_A_ receptors containing δ- and α4-subunits is not altered and the GABAergic neurotransmission is preserved. Indeed, our RT-PCR experiments revealed enhanced expression of the mRNA encoding the α4-subunit in patients without anxiety and/or depression, a situation that could be related to enhanced tonic inhibition or may merely reflect compensatory changes.

[^3^H]-Flunitrazepam is a BDZ that labels GABA_A_ receptors containing γ2-subunit in combination with α1–3 subunits and are responsible for mediating phasic inhibition at synaptic sites (Lavoie and Twyman, 1996). BDZ can functionally potentiate the effects induced by sub-maximal concentrations of GABA by enhancing GABA affinity (Olsen, 1981). Previous studies in patients with TLE indicate an increased expression of γ2-subunit (Sperk et al., 2009), whereas the BDZ binding results are controversial (la Fougère et al., 2009). From our results, it seems that patients with TLE and mood disorders demonstrate absence of changes in BDZ binding in the temporal neocortex, but an upregulation of γ2-subunit. It remains to be elucidated whether, in these patients, the upregulation of γ2-subunit in temporal neocortex is primary or is secondary to the disease, and represents a compensatory response to the GABA deficit. Concerning patients with TLE without anxiety and depression, we found augmented BDZ binding and increased expression of γ2-subunits. This situation can be associated with fast synaptic GABA-induced inhibition and reduction in anxiety-like and depressive-like behaviors (Earnheart et al., 2007; Vithlani et al., 2013).

In agreement with the increased expression of the α4- and γ-subunits, and the absence of changes in GABA_A_/BDZ binding found in the present study, results obtained from other authors using resected hippocampal tissue from patients with TLE support the idea that the inhibition mediated by GABAergic system is conserved or even augmented (Babb et al., 1989; Mathern et al., 1995). However, an important condition to be considered is that a low ambient GABA represents an inadequate situation to activate GABA_A_ receptors mediating tonic inhibition with a consequent facilitation of seizure activity, depression-like, and anxiety-like behaviors (Merali et al., 2004; Maguire and Mody, 2008; Hines et al., 2012). Our experiments revealed reduced GABA tissue levels in the neocortex of patients with TLE and comorbid anxiety and depression, a situation that can be associated with the low GABA release in pharmacoresistant epilepsy described by other authors (During and Spencer, 1993; Luna-Munguia et al., 2011).

Activation of GABA_B_ receptors starts several signaling cascades at pre- and postsynaptic levels essential for the stability of the cortical network activity and modulation of gamma oscillations essential for cognitive processes (Kohl and Paulsen, 2010). In contrast, a deficiency in the neurotransmission mediated by GABA_B_ receptors has been associated with changes in cortical gamma oscillations with consequent psychiatric disorders such as schizophrenia (Uhlhaas and Singer, 2010). We found that patients with TLE without anxiety and/or depression presented decreased GABA_B_-induced G-protein activation restricted to layers V–VI. In contrast, patients with TLE and comorbid anxiety and/or depression showed reduced GABA_B_ receptor-induced G-protein activation in all cortical layers. These findings associated with low GABA tissue content suggest a deficit in the neurotransmission induced by GABA_B_ receptors in temporal cortex of patients with TLE and comorbid anxiety and/or depression, a situation similar to that found in patients with mood disorders (Enna and Bowery, 2004; Croarkin et al., 2011). Indeed, the high GABA_B_ binding found in cortical layers III–IV and V–VI (present study), as well as the up-regulation of GABA_B_ in remaining neurons in hippocampus (Princivalle et al., 2002) can represent a compensatory mechanism resulting from the deficient neurotransmission mediated by these receptors in patients with TLE.

The comorbidity of mood disorders in patients with epilepsy has been associated with a higher perception of side effects of the therapy with AEDs (Gómez-Arias et al., 2012). AEDs therapy can induce uncoupling of GABA/BZD site interactions and alterations in GABA_A_ and GABA_B_ function (Mula and Sander, 2007); these changes may result from the repetitive administration of BDZs and/or AEDs that enhance GABA exposure such as tiagabine and vigabatrin (Suzuki et al., 1991; Gravielle et al., 2005; Pericić et al., 2007). Carbamazepine and valproic acid increase the number of GABA_B_ binding sites in the rat hippocampus when applied chronically, an effect that has been associated with the mood stabilizing effects induced by these drugs (Motohashi, 1992). In contrast, long-term exposure to these AEDs may decrease the GABA_B_ receptor function, an effect analogous to that produced by the subchronic administration of baclofen (Pacey et al., 2011). Our results do not support a significant association between the pharmacotherapy with AEDs inducing GABAergic effects in temporal neocortex and the comorbid anxiety and/or depression in patients with TLE. However, future studies including other brain areas and a larger number of patients should be carried out to support this hypothesis.

Affective disorders have been associated with disturbances in the second messenger signaling via G-protein function (Pacheco et al., 1996). Hyperfunction of G proteins leads to characteristics of a manic or depressive state caused by instability in the activities of protein kinases C (Avissar and Schreiber, 1992), a family of enzymes that are involved in the signal transduction mechanism of the GABA_B_ receptors (Taniyama et al., 1992). This study indicates an imbalance of the G-protein activation induced by stimulation of GABA_B_ receptors in the temporal neocortex of patients with TLE and comorbid anxiety and/or depression. This situation should be kept in mind when considering these receptors as promising targets for the therapy of psychiatric disorders associated with TLE.

The comorbidity of mood disorders in TLE has also been related to a higher seizure frequency (Grabowska-Grzyb et al., 2006; Mula and Sander, 2007; Peng et al., 2014) and longer duration of active epilepsy (Gonçalves and Cendes, 2011). Our results revealed that the lower values for BDZ binding, GABA_B_-induced G-protein activation, and GABA tissue content correlated with a higher seizure frequency and the longer duration of epilepsy of patients with TLE and comorbid anxiety and/or depression. In contrast, the higher BDZ binding and elevated GABA_B_-induced G-protein activation correlated with a lower seizure frequency of patients with TLE without psychiatric disturbances. It is possible that the higher seizure frequency and longer duration of epilepsy augment the exposure to elevated extracellular GABA levels during the ictal period. This situation may result in GABA_A_ desensitization, dysregulation of the neurotransmission mediated by GABA_B_ receptors, and uncoupling of GABA/BZD site interactions (Gravielle et al., 2005), conditions that could facilitate the comorbid anxiety and/or depression of patients with TLE. Finally, further experiments should be carried out to identify if the receptor binding alterations detected in the present study are the consequence of changes in the number or affinity of the receptors evaluated. We also suggest future studies in patients with anxiety and depression disorders but without epilepsy to explain the findings obtained in the present study.

## Author Contributions

The author Luisa Rocha designed the study and prepared the manuscript. The author Mario Alonso-Vanegas performed the epilepsy surgery to all the patients. The author Iris E. Martínez-Juárez carried out the pre-surgical evaluation of the patients with pharmacoresistant epilepsy. The authors Sandra Orozco-Suárez, David Escalante-Santiago, and Iris Angélica Feria-Romero performed the semiquantitative RT-PCR analysis. The author Cecilia Zavala-Tecuapetla carried out the functional autoradiography experiments. Under the supervision of Mario Alonso-Vanegas, the authors José Miguel Cisneros-Franco, Ricardo Masao Buentello-García, and Jesús Cienfuegos analyzed the clinical data of the patients and their correlation with the results obtained. All authors contributed to manuscript revisions and have approved the final manuscript.

## Conflict of Interest Statement

The authors declare that the research was conducted in the absence of any commercial or financial relationships that could be construed as a potential conflict of interest.

## Supplementary Material

The Supplementary Material for this article can be found online at http://www.frontiersin.org/Journal/10.3389/fncel.2014.00442/abstract
