# Supplementary material for: GABAergic Alterations in Neocortex of Patients with Pharmacoresistant Temporal Lobe Epilepsy Can Explain the Comorbidity of Anxiety and Depression: The Potential Impact of Clinical Factors
Source: Front Cell Neurosci. 2015 Jan 5;8:442. doi: 10.3389/fncel.2014.00442 (PMC4283637; doi:10.3389/fncel.2014.00442)
Supplement: Supplementary file 1 [file Table_1.DOC]

# **TABLE 1.** Summary of clinical data from patients with temporal lobe epilepsy, tumor, lesion and autopsies

| **Patient** | **Gender** |  | **Age**  **(years)** | **Seizure onset age (years)** | **Precipitating factors** | **Side of focus**  **or**  **lesion** | **Duration of epilepsy**  **(years)** | **Seizure**  **frequency**  **(month)** | **AEDs**  **during epilepsy** | **AEDs**  **before surgery** | **Psychiatric comorbidity** | **Surgical outcome**  **(Engel´s classification)** | **Final**  **diagnosis or cause of death**  **(PMI in hours)** |
| --- | --- | --- | --- | --- | --- | --- | --- | --- | --- | --- | --- | --- | --- |
| **P81** | F |  | 38 | 6 | No | Left | 32 | 3 | DFH, VAP, CBZ, OXCBZ, LMG, LVT | OXCBZ | Depression and anxiety  of 4 years of evolution and treated with antidepressive drugs | Class II | MTLE and HS |
| **P93** | M |  | 25 | 7 | Hypoxia | Left | 18 | 16 | GBP, LVT, CNZ, LMG, PMD, CBZ, VAP, DFH, LMG | CBZ, CNZ  PMD | Diagnosis of depression  9 months before the surgery and treated with antidepressive drugs | Class I | MTLE and HS |
| **P95** | F |  | 47 | 25 | Cerebral thrombosis | Right | 22 | 14 | DFH, AVP, CBZ, PMD, LMG, TOP, CNZ, CLB, LVT, ZNS | LMG, CNZ, ZNS | Presurgical diagnosis of depression | Class I | MTLE and HS |
| **P98** | M |  | 32 | 8 | Hypoxia | Left | 24 | 7.5 | PHE, CLB, TOP, LMG, PMD, CBZ, VAP, DFH | VAP, LMG, CLB | Presurgical diagnosis of depression | Class IV | MTLE and HS |
| **P99** | F |  | 48 | 46 | No | Left | 2 | 18 | VAP, CNZ, LVT, DZP | VAP, CNZ | Depression and anxiety  of 3 years of evolution and treated with antidepressive drugs | Class I | Cavernoma in hippocampus and parahippocampus |
| **P105** | M |  | 24 | 6 | Hypoxia,  febrile seizures  in childhood | Right | 18 | 4 | CLB, CNZ, TOP, CBZ, VAP | VAP, TOP  CNZ | Presurgical diagnosis of depression | Class I | MTLE and HS |
| **P107** | M |  | 34 | 6 | Cerebral injury | Left | 28 | 3 | DFH, VAP, CBZ, PMD, CNZ, LVT | VAP, CBZ, CLB, AZM | Depression and anxiety  of 6 years of evolution and treated with antidepressive drugs | Class II | MTLE and HS |
| **P119** | M |  | 32 | 28 | No | Left | 4 | 1 | DFH | PHE | Presurgical diagnosis of depression | Class i | Oligoastrocytoma in superior temporal gyrus |
| **P125** | M |  | 45 | 17 | No | Left | 28 | 48 | CLB, LVT, TOP, DFH, OXCBZ | CLB, TOP, OXCBZ | Presurgical diagnosis of anxiety | Class I | MTLE and HS |
| **P127** | F |  | 38 | 3 | Cerebral injury,  febrile seizures  in childhood | Left | 35 | 15 | DFH, VAP, CBZ, PHE, OXCBZ, LMG, CNZ, CLB, DZP, AZM | VAP, LMG  OXCBZ | Presurgical diagnosis of depression  and anxiety | Class I | MTLE and HS |
| **P37** | F |  | 27 | 11 | No | Right | 16 | 32 | DFH, CBZ, PMD | PHE, CBZ, PMD | No | Class III | Astrocytoma  in occipitotemporal gyrus |
| **P56** | M |  | 27 | 4 | Hypoxia,  febrile seizures  in childhood | Right | 23 | 3 | DFH, VAP, CBZ, LMG, CNZ, PHE | CBZ, LMG,,  CNZ | No | Class I | MTLE and HS |
| **P54** | F |  | 34 | 1.3 | Cerebral injury | Right | 32.6 | 38 | DFH, VAP, CBZ, LMG, TOP | CBZ, LMG | No | Class II | MTLE and tumor |
| **P83** | F |  | 35 | 0.16 | No | Right | 0.16 | 5 | DFH, VAP, CBZ | CBZ, VAP | No | Class I | MTLE and HS |
| **P84** | F |  | 29 | 1 | No | Right | 1 | 5 | DFH, VAP, CNZ | VAP, CNZ | No | Class I | MTLE and HS |
| **P88** | F |  | 29 | 8 | No | Right | 21 | 30 | CNZ, LVT, GBP, DFH | DFH | No | Class I | MTLE and HS |
| **P92** | F |  | 27 | 27 | No | Left | 0.33 | 1 | DFH, VAP, CBZ, PMD, LMG, CNZ, LVT, GBP | DFH, VAP | No | Class I | Oligoastrocytoma in superior temporal gyrus |
| **P103** | M |  | 28 | 13 | No | Right | 15 | 3 | PHE, CLB, PMD, LMG, DFH, VAP, CBZ | LMG, CBZ | No | Class I | Glioma in hippocampus and parahippocampus |
| **P104** | M |  | 60 | 6 | No | Left | 54 | 9 | VAP, CBZ, TOP, CNZ, CLB | VAP, TOP, CNZ | No | Class I | MTLE and HS |
| **P112** | M |  | 40 | 40 | No | Right | 0.16 | 1 | DFH | DFH | No | Class I | Oligoastrocytoma in superior temporal gyrus |
| **P116** | F |  | 24 | 10 | No | Left | 14 | 102 | VAP, CBZ, LMG | CBZ | No | Class I | Neuroectodermal tumor in temporal pole |
| **P123** | M |  | 35 | 13 | Cerebral injury | Left | 22 | 12.5 | LMG, CBZ, DFH | VAP, CBZ, LMG | No | Class I | MTLE and HS |
| **P124** | F |  | 52.9 | 52.8 | Tumor | Left | 0.1 | 4 | DFH | DFH | No | Class I | Epidermoid carcinoma in temporal cortex |
| **P142** | F |  | 35 | 3 | No | Left | 3 | 5 | CBZ | CBZ | No | Class I | MTLE and HS |
| **P144** | F |  | 28 | 17 | No | Right | 11 | 7 | VAP, CBZ, LMG, TOP, | LVT | No | Class I | MTLE and HS |
| **P148** | M |  | 24 | 14 | No | Right | 10 | 3 | DFH | LMG, VAP | No | Class I | Oligoastrocytoma  in temporal cortex |
| **P102** | M |  | 57 | -- | -- | Left | -- | -- | -- | -- | -- | -- | Glioblastoma multiforme in T2 gyrus |
| **P113** | F |  | 28 | -- | -- | Right | -- | -- | -- | -- | -- | -- | Glioma in T4 gyrus |
| **P114** | M |  | 28 | -- | -- | Left | -- | -- | -- | -- | -- | -- | Astrocytoma in T1-T3 gyri |
| **P121** | M |  | 37 | -- | -- | Left | -- | -- | -- | -- | -- | -- | Oligoastrocytoma in parieto-temporal cortex |
| **P141** | M |  | 25 | -- | -- | Right | -- | -- | -- | -- | -- | -- | Glioma in parieto-temporal cortex |
| **P145** | F |  | 63 | -- | -- | Left | -- | -- | -- | -- | -- | -- | Astrocytoma temporo-insular |
| **C1** | M |  | 40 | -- | -- | -- | -- | -- | -- | -- | -- | -- | Asphyxia  (5) |
| **C2** | M |  | 29 | -- | -- | -- | -- | -- | -- | -- | -- | -- | Hypovolemic shock  (5) |
| **C3** | M |  | 37 | -- | -- | -- | -- | -- | -- | -- | -- | -- | Hypovolemic shock  (10) |
| **C4** | M |  | 33 | -- | -- | -- | -- | -- | -- | -- | -- | -- | Hypovolemic shock  (14) |
| **C5** | M |  | 47 | -- | -- | -- | -- | -- | -- | -- | -- | -- | Lymphoma  (2) |
| **C6** | M |  | 51 | -- | -- | -- | -- | -- | -- | -- | -- | -- | Pulmonary cancer  (4) |

AED, Antiepileptic Drugs; AZM, Acetazolamide; CBZ, Carbamazepine; CLB, Clobazam; CNZ, Clonazepam; DFH, Diphenylhydantoin, DZP, Diazepam; F, Female; GBP, Gabapentin; HS, Hippocampal Sclerosis; LMG, Lamotrigine; LVT, Levetiracetam; M, Male; MTLE, Mesial Temporal Lobe Epilepsy; OXCBZ, Oxcarbazepine; PHE, Phenobarbital; PMD, Primidone; PMI, Postmortem Interval; VAP, Valproic Acid; TOP, Topiramate; ZNS, Zonizamide.
